# Recurrence of keratinocyte cancers after superficial radiation therapy

**DOI:** 10.1093/skinhd/vzag047

**Published:** 2026-05-14

**Authors:** Laura Daley, Kurt Byrnes, Peter J Caldwell, James W Wells, Colin Dicks, Helmut Schaider

**Affiliations:** Faculty of Health Sciences and Medicine, Bond University, Gold Coast, QLD, Australia; JustSkin, Maroochydore, QLD, Australia; JustSkin, Maroochydore, QLD, Australia; Frazer Institute, University of Queensland, Brisbane, QLD, Australia; JustSkin, Maroochydore, QLD, Australia; JustSkin, Maroochydore, QLD, Australia; Frazer Institute, University of Queensland, Brisbane, QLD, Australia; Department of Dermatology, State Hospital Klagenfurt, Klagenfurt, Carinthia, Austria

## Abstract

Superficial radiation therapy (SRT) is a noninvasive treatment that uses low-energy X rays (50–150 kV) to treat skin cancers. SRT is used by dermatologists due to its accessibility and safety profile. Higher-energy radiation therapies are typically used by radiation oncologists. This narrative review aimed to evaluate the long-term clearance rates of keratinocyte cancers (KCs) treated with SRT. A literature search of MEDLINE, PubMed and Embase was performed in May 2024 and identified 12 studies that used SRT to treat KCs. The 12 studies included 6027 patients and reported a total of 9157 lesions treated with SRT. All studies were of a retrospective cohort design. No prospective cohort studies or randomized controlled trials (RCTs) were available for inclusion. Patients with KCs treated with SRT had a mean recurrence rate of 4.6% at the 3-year follow-up, with longer follow-up durations linked to higher recurrence rates (*R*² = 0.486). Recurrence rates for squamous cell carcinomas and basal cell carcinomas treated with SRT were 5.0% and 3.9%, respectively. The outcomes for SRT are encouraging; however, with the lack of head-to-head trial data in the form of RCTs, the evidence is of poor quality. These results justify the consideration of prospective studies, including RCTs, to compare the efficacy and safety of SRT with other established approaches. While SRT shows promise as a treatment option for KCs, the limited strength of the available evidence makes this conclusion uncertain.

## Teaser

Superficial radiation therapy (SRT) is a noninvasive, low-energy X-ray treatment sometimes used by dermatologists to manage keratinocyte cancers. A narrative review of 12 retrospective studies involving 6027 patients and 9157 lesions reported a mean recurrence rate of 4.6%, with higher rates seen over longer follow-up periods. However, the evidence is limited by the retrospective design of all studies and the lack of randomized controlled trials, making the long-term effectiveness of SRT uncertain.

What is already known about this topic?

- Long-term outcome data for keratinocyte cancer (KC) treatments, particularly superficial radiation therapy (SRT), are limited.
- SRT targets lesions with low-penetration radiation (50–150 kV), minimizing damage to underlying tissues.
- SRT is commonly used by dermatologists due to its accessibility and safety profile, unlike higher-energy radiation therapies, which are typically used by radiation oncologists.

What does this study add?

- This is the first narrative review to examine the use of SRT in the treatment of KCs, providing a consolidated summary of the existing evidence for its use.
- This study provides evidence that SRT offers long-term local control of KCs, but the evidence available is of low quality.
- SRT is highlighted as a noninvasive and effective treatment option, especially for patients who are not ideal candidates for surgery.

Australia is in the unenviable position of having the highest prevalence of skin cancer in the world.^1^ Risk factors for skin cancer include sun exposure, increasing age, lighter skin phototype, genetic predisposition and immunosuppression.^2–4^ Basal cell carcinomas (BCCs) account for approximately 70% of keratinocyte cancer (KC) cases, and squamous cell carcinomas (SCCs) around 30%.^5^ Approximately 50% of BCC recurrences occur within the first year of treatment and approximately 80% within 5 years.^6^ With regard to SCCs, 70–80% recur within 2 years of treatment, while approximately 95% recur within 5 years.^7^

Treatment modalities for KCs include surgical excision, electrodessication and curettage, cryotherapy, Mohs micrographic surgery (MMS), topical creams, photodynamic therapy and oral medications like vismodegib.^8–11^ Radiotherapy has been widely used for many decades to treat skin cancer.^12^ Radiotherapy modalities include superficial radiation therapy (SRT), orthovoltage radiation, electrons and Co60 photons, brachytherapy and external beam radiation therapy.^13^

Based on its growing use among dermatologists, SRT was selected as the focus for this review.^14^ Other forms of radiation therapy with higher kilovoltage (kV) settings are often preferred by radiation oncologists to treat deeper tissues.^15^ However, SRT only penetrates skin up to 1 cm, leaving deeper structures intact and unharmed, and making it safe for dermatological practices.^16,17^ SRT is a painless, nonsurgical treatment modality for KCs, often used by dermatologists in the office setting.^16^ A 50–150-kV X-ray machine is used, which generates low-energy photons that are mostly absorbed by 5-mm of skin tissue.^17^ In the early twentieth century, the original superficial radiation machines were in widespread use. However, a rise in enthusiasm for dermatological surgery, as well as topical and light-based therapies, saw it fall out of favour.^18,19^

While there are numerous reviews of SRT, there is a notable lack of information on recurrence rates following its use to treat skin cancer. This review aimed to evaluate the available data relating to the long-term clearance rates of KCs treated with SRT to provide clearer insights into the effectiveness and safety profile of SRT to better inform clinical decision-making.

## Materials and methods

### Literature search

We conducted a narrative review, a type of review that summarizes limited research on a topic without the strict methods or rigour of a systematic review. Accordingly, the literature search aimed to capture relevant studies broadly rather than through predefined systematic criteria. The review was registered with Open Science Framework (https://osf.io/9u83x). A search of PubMed, MEDLINE and Embase was performed for studies within the scope of the review. The reference lists of relevant studies were manually checked for additional articles that were missed from the initial search. The primary aim of the review was to analyse the treatment outcome, which was defined as complete clearance of the KC without recurrence. The secondary aim was to analyse adverse events (AEs) associated with SRT.

The search terms were as follows: (‘keratinocyte cancer’ OR ‘non melanoma skin cancer’ OR ‘basal cell carcinoma’ OR ‘BCC’ OR ‘cutaneous squamous cell carcinoma’ OR ‘SCC’ OR ‘non melanoma skin cancer’) AND (‘superficial radiation’ OR ‘superficial radiotherapy’ OR ‘ultrasoft radiation’ OR ‘ultrasoft radiotherapy’ OR ‘Grenz rays’ OR ‘bucky rays’).

**Figure 1:**
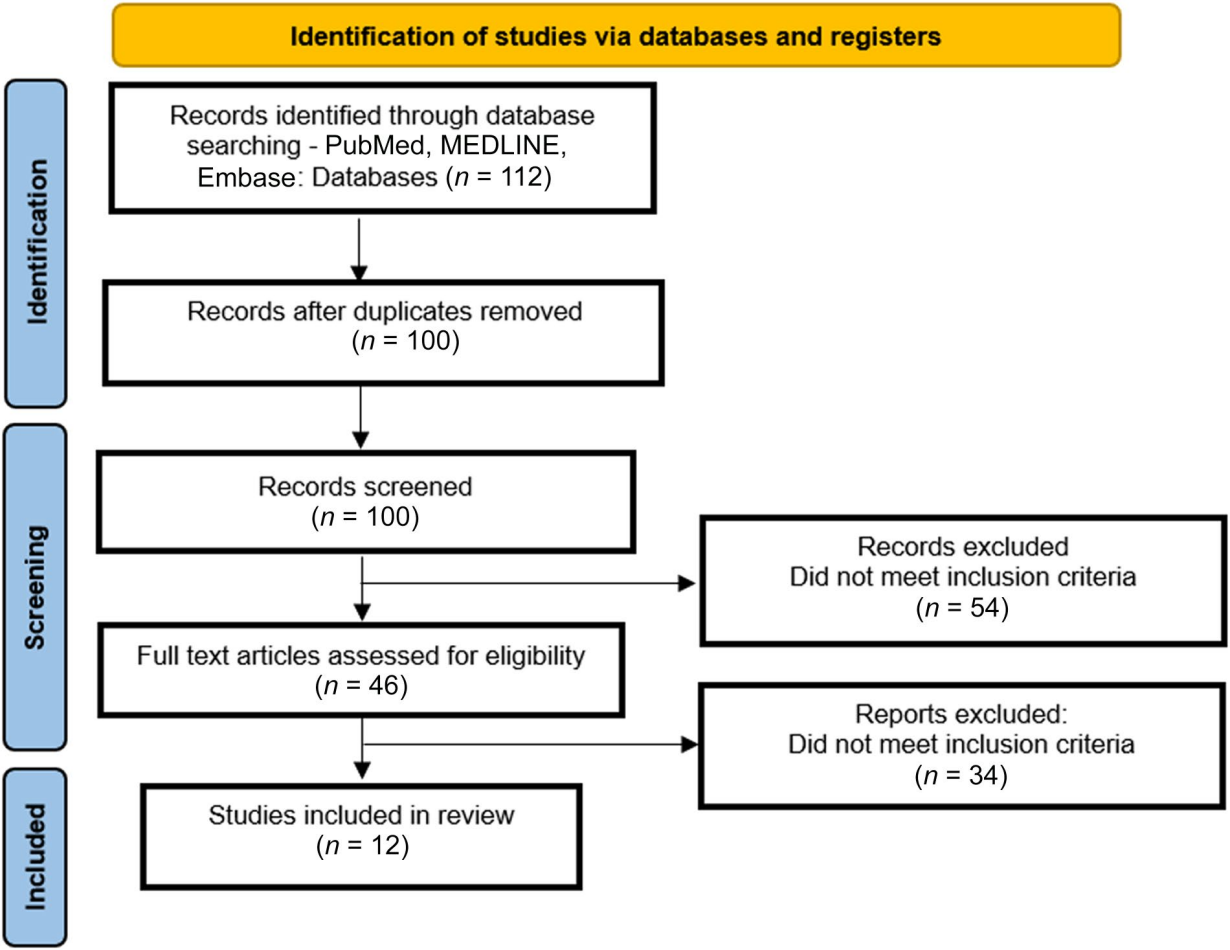
Flow diagram of the literature search.

A total of 112 records were identified via the database search (Figure 1). After removing duplicates, 100 articles were screened by title and abstract. Of these, 54 were excluded due to lack of relevance given that they did not align with the specific research focus, lacked methodological rigour or addressed topics outside the scope of the study. The inclusion criteria were articles written in English and explored SRT of KC. Studies were required to have published recurrence rates, voltage and dose of SRT used, follow-up durations (median or mean), and detailed descriptions of patient selection and treatment technique. Articles without original data, including reviews, were excluded. Case reports were also excluded.

Full-text assessment of the remaining 46 articles resulted in the exclusion of 34 due to them not meeting the inclusion criteria. Ultimately, 12 retrospective studies were included. No randomized controlled trials (RCTs) were available for inclusion.

### Presentation of findings and data analysis

The ﬁndings from the literature review were summarized into standardized tabulated forms and included the following key data for each of the studies: authors, number of clinics, number of patients, age of patients, number of lesions, predominant site of lesions, treatment modalities, follow-up periods, recurrence rates, number of fractions, total dosage and AEs. Recurrence rate was the key outcome. For some studies, local control rates were converted into recurrence rates to facilitate comparison. The mean recurrence rate was calculated using all recurrence rates.

### Statistical analysis

Jamovi was used to formulate a correlation scatterplot (https://www.jamovi.org/) and to analyse recurrence rates.

## Results

Twelve studies were included in the final review (Table S1; see Supporting Information).^20–31^ Combining individual studies produced a cohort of 6027 patients mainly from Europe and the USA. All 12 studies were retrospective cohort studies, with the majority being single-institutional studies. However, the studies by Moloney *et al*.^22^ and Roth *et al*.^27^ were multi-institutional, with eight and four clinics included, respectively. Most of the studies only included patients who had undergone SRT. Locke *et al*.^30^ compared SRT treatments with electron beam irradiation or a combination of electron beam irradiation and SRT.

As shown in Table S2 (see Supporting Information), most studies (*n* = 8) included biopsied lesions.^20,21,23,26,28–31^ Three studies did not specify whether lesions were biopsied,^22,25,27^ and one reported a biopsy rate of 53%.^24^ Only one study noted the immuno­suppression status of patients.^20^ Lesions were mainly BCC or SCC. Most involved primary lesions, although patients with recurrences or treated with an adjuvant were mentioned in four papers.^21,24,30,31^ Multiple lesions per patient were often treated. The head and neck were the most common sites. One study focused on the vermilion border,^21^ one on the lower extremities^28^ and one did not specify.^26^

### Recurrence rates

Overall, the average local recurrence rate for KCs following SRT was 4.6%. The mean (SD) follow-up period was 38.9 (0.86) months. This was calculated from the 10 studies where this parameter was reported.^20–22,25–31^ Two studies reported follow-up times as median values.^23,24^ Attempts to obtain mean data from the authors of these studies were unsuccessful, leading to their exclusion from the linear regression model.

**Figure 2:**
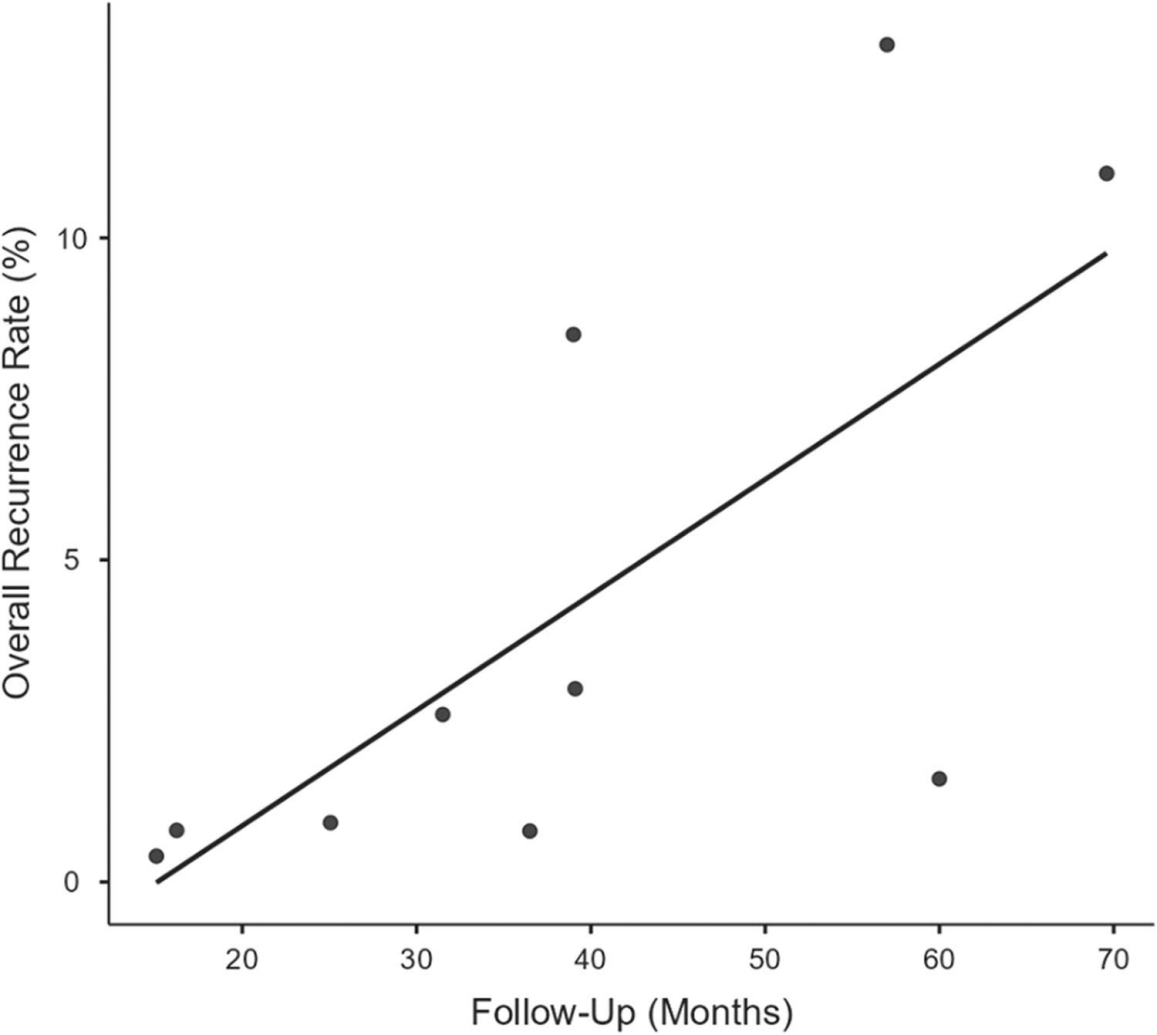
Superficial radiation therapy linear regression model: mean duration of follow-up (months) vs. recurrence rate (%).

A regression model was derived from 10 of the 12 articles that reported mean follow-up duration and examined the relationship between recurrence rate and follow-up duration (Figure 2). The regression model showed a positive correlation: the longer the mean follow-up period, the greater the recurrence rate of KCs treated with SRT. An *R*^2^ value of 0.486 indicated that approximately 48.6% of the variability in the recurrence rate was explained by the follow-up period in the model. In relation to SRT of KCs, it was found that studies with more participants appeared to have lower recurrence rates (Table S3; see Supporting Information). For example, the three studies with the highest number of participants were those of Moloney *et al*. (*n* = 1709), Tran *et al*. (*n* = 1243) and Cognetta *et al*. (*n* = 1149),^22,25,29^ which reported overall recurrence rates of 0.9%, 0.4% and 2.6%, respectively.

In addition, it was noted that some studies that reported higher recurrence rates, such as those of Zagrodnik *et al*. (12.6%) and Locke *et al*. (11%),^23,30^ used relatively lower total dosages of SRT vs. the treatment protocols used in all of the other studies (Table S3).^20–22,24–29,31^ Overall, the recurrence rate for head-and-neck KCs treated with SRT was 4.8%.^20,22–25,27,29–31^ Lip lesions treated with SRT had a recurrence rate of 9%, while the recurrence rate of KCs of the lower extremities treated with SRT was 3%.^21,28^

### Energy and dose

A diverse range of dose prescriptions and fractionation schedules were applied in the treatment of KCs with SRT, reflecting the heterogeneity in clinical practice and patient needs (Table S1). SRT was prescribed in 2–30 fractions with a total dose of 2–60 Gy.

### Adverse events

Many studies did not adequately cover AEs associated with treatment; however, reported events included erythema, dry and moist desquamation, ulceration, local mild mucositis and epiphora (watery eyes; after treatment of an eyelid lesion).

## Discussion

This review evaluates the recurrence rates of KCs treated with SRT and attempts to fill a gap in the literature. Overall, the analysis of 12 studies, comprising 6027 patients, provided a mean KC recurrence rate of 4.6% across various treatment sites with a mean (SD) follow-up duration of 38.9 (0.86) months. It was also found that longer follow-up periods were linked to higher recurrence rates (*R*^2^ = 0.486).

The review highlights that recurrence rates of KCs treated with SRT may vary depending on a lesion’s location, with lip cancers showing a higher recurrence rate (9%) than those on the lower extremities (3%). This is consistent with research showing that KCs on the lip are at higher risk of recurring.^32^ Studies with larger cohorts showed lower KC recurrence rates, suggesting more ­reliable estimates due to comprehensive data collection, reduced ­variability or improved treatment protocols. In addition, some studies reporting high recurrence rates used a relatively lower total dosage of SRT. This indicates that adequate dosing is crucial for treatment effectiveness.

The retrospective and nonrandomized nature of the included studies overall means that direct comparisons of KC recurrence rates following SRT and conventional treatments such as surgical excision and MMS cannot be made. Multiple factors influence recurrence rates, including tumour size, depth, histological subtype, anatomical location, and whether the lesion is primary or recurrent. The tumours probably selected for SRT are of predominantly indolent types, with MMS generally being reserved for higher-risk tumours, thereby making direct comparisons difficult. SRT may generally be chosen for older patients who, on occasion, have inoperable lesions.

This study addresses a gap in the literature by focusing on recurrence rates following SRT, which is less well studied than standard surgical excision and MMS. Much of the existing literature focuses on the outcomes of surgical excision, with recurrence rates influenced by factors such as tumour margins and patient risk profiles. This approach provides a more detailed understanding of the effectiveness of SRT not only as an adjunct to surgery, but also as a potential first-line treatment.

This review has several strengths. The analysis of follow-up duration highlights the critical role of this period in detecting recurrences, showing a positive correlation between longer follow-up durations and higher recurrence rates. This emphasizes the importance of extended monitoring for accurate assessment of the long-term efficacy of SRT. In addition, this is the first narrative review, to our knowledge, to focus specifically on the use of SRT in the treatment of KCs, providing a consolidated overview of the available evidence.

Several weaknesses that impact the strength of the conclusions must be mentioned. All studies of SRT included in this review were retrospective cohort studies. No prospective studies or RCTs of SRT vs. standard surgical approaches were included. Important details on the characteristics of the included KCs were not available for inclusion, particularly tumour size, whether invasion had occurred, whether patients were immunosuppressed and whether patients had a history of previous recurrent tumours. Another significant issue is the heterogeneity in outcome measurement across the included studies. Endpoints such as local control, cosmetic outcomes, AEs and recurrence rates are often inconsistently defined and reported, and, unlike surgery, SRT is not accompanied by histological confirmation of complete tumour removal, making immediate evaluation impossible. The variability in SRT protocols, including differences in dose prescription, fractionation and equipment, further complicates standardization across studies. Variation in follow-up durations makes further comparison of results difficult.

### Future research directions

Future studies should focus on RCTs that directly compare SRT with surgical excision and MMS for the treatment of KCs. However, SRT is typically reserved for patients deemed unsuitable for surgery, making randomization difficult and introducing bias, limiting the generalizability of findings. Additionally, extended follow-up periods are essential to address the current lack of robust long-term data. In the studies of SRT included in this review, shorter follow-up periods (20–30 months) may underestimate the risk of KC recurrence, while extended follow-up durations (60–70 months) show recurrence rates exceeding 10%. This highlights the need for long-term monitoring to assess treatment efficacy accurately. Patients are often lost to long-term follow-up due to access and resource issues, and this is even more common in older and frail patients. Resources should be put in place to facilitate the ability of older and frail patients to attend long-term follow-ups and be monitored over longer periods.^24^

Research is needed to investigate the long-term outcomes and safety profile of SRT in treating KCs, with a particular focus on documenting and analysing AEs. Systematic assessment of the frequency, severity and management of AEs, and understanding strategies to minimize risks, may lead to improved patient outcomes.

### Conclusions

This narrative review found that the recurrence rate of KCs treated SRT is encouragingly low, but further studies are needed to establish where this approach fits in with other, more established treatment options. Patients with KCs treated with SRT had a mean recurrence rate of 4.6% over approximately 3 years, with longer follow-ups linked to higher recurrence rates. The recurrence rates of SCCs and BCCs treated with SRT were 5.0% and 3.9%, respectively. Contemporary evidence for the treatment of KCs with SRT is predominantly based on retrospective studies, with no RCT data available. SRT shows promise as a treatment option for KCs, but the low strength of the available evidence limits the certainty of this conclusion. As more reliable evidence becomes available on the outcomes of SRT in the treatment of KCs, as well as recurrence rates after treatment, its role as a possible therapeutic option for KCs may warrant further consideration.

## Supplementary Material

vzag047_Supplementary_Data
