# Supplementary material for: Recurrence of keratinocyte cancers after superficial radiation therapy
Source: Skin Health Dis. 2026 May 14;6(4):369–74. doi: 10.1093/skinhd/vzag047 (PMC13425004; doi:10.1093/skinhd/vzag047)
Supplement: vzag047_Supplementary_Data [file vzag047_supplementary_data.zip › Table S1.docx]

**Table S1 Overview of SRT for the treatment, techniques, and adverse effects in relation to SRT for the treatment of keratinocyte cancer.**

| **Sources** | **Number of clinics** | **Treatment**  **Dates** | **Number of patients** | **Age**  **(years)** | **Total Number of Keratinocyte Cancers^a^** | **Number of fractions^b^** | **Total**  **Dosage^b^** | **Adverse**  **Events** |
| --- | --- | --- | --- | --- | --- | --- | --- | --- |
| Barysch *et al* ^32^ | 1 | 1960-2004 | 179 | 69 (mean) | Total = 180  [SCC=180]  [BCC=N/A] | Mean: 11.4 | Mean: 48.2 Gy | N/A |
| Piccinno *et al* ^30^ | 1 | 1974-2015 | 47 | 70 (median) | Total = 47  [SCC=35]  [BCC=12] | Mode:11 | Mean: 57 Gy | Local mild mucositis |
| Moloney *et al* ^23^ | 8 | 2017-2020 | 1709 | 74.04 (median) | Total = 3050  [SCC=924]  [BCC=1460] | Median: 20 | Mean: 52.16 Gy | N/A |
| Zagrodnik *et al* ^29^ | 1 | 1981 - 1991 | 148 | 69 (median) | Total = 175  [SCC=N/A]  [BCC=175] | Range: 5-30 | Range: 2-8 Gy | N/A |
| Thom *et al* ^31^ | 1 | 1999-2001 | 259 | 76 (median) | Total = 369  [SCC=369]  [BCC=237] | Mode: 6 | Mode: 36 Gy | Epiphora after eyelid lesion treatment |
| Tran *et al* ^27^ | 1 | 2016-2022 | 1243 | 73.2 (mean) | Total = 1899  [SCC=467]  [BCC=981] | Mean: 20.2 | Mean: 53.64 Gy | Erythema, dryness, ulceration |
| Yu *et al* ^26^ | 1 | 2017-2018 | 93 | 69 (median) | Total = 133  [SCC=17]  [BCC=67] | Median: 20 | Median: 51.28 Gy | N/A |
| Roth *et al* ^24^ | 4 | prior to Jan 2015 | 516 | 79 (mean) | Total = 776  [SCC=328]  [BCC=448] | Mean: 12.3 | Mean: 46.52 Gy | N/A |
| Roth *et al* ^33^ | 1 | 2011-2014 | 105 | 82.5 (mean) | Total = 151  [SCC=113]  [BCC=38] | Mode: 15 | Mean: 50.19 Gy | Red skin, ulcerated skin, oedema, itchiness. |
| Cognetta et al ^28^ | 1 | 2000-2010 | 1149 | 79 (mean) | Total = 1715  [SCC=994]  [BCC=712] | 5 [standardised] | 35 Gy [standardised] | N/A |
| Locke *et al* ^25^ | 1 | 1966-1977 | 468 | 72.5 (median) | Total = 531  [SCC=142]  [BCC=389] | Range: 2-11 | Range: 6-60 Gy | N/A |
| Madorsky *et al* ^21^ | 1 | 2012-2018 | 111 | Not Included | Total = 131  [SCC=22]  [BCC=104] | Mean: 13 | Mean: 45.44 Gy | Skin weeping, crusting, minor bleeding. |

^a^: the total number of keratinocyte cancers includes cancers other than BCCs and SCCs.

^b^: for number of fractions and total dose, there was lack of consistency among studies in terms of how these statistics were reported. Preferably, the mean was used. However, the mode (most frequently used) and range were sometimes used due to lack of available data.
