# Supplementary material for: Recurrence of keratinocyte cancers after superficial radiation therapy
Source: Skin Health Dis. 2026 May 14;6(4):369–74. doi: 10.1093/skinhd/vzag047 (PMC13425004; doi:10.1093/skinhd/vzag047)
Supplement: vzag047_Supplementary_Data [file vzag047_supplementary_data.zip › Table S2.docx]

**Table S2 Overview of patient and lesion details, and outcomes in relation to SRT for the treatment of keratinocyte cancer.**

| **Sources** | **Biopsied** | **Histopathological classification** | **Diameter (best available data used)** | **Immunosuppression** | **Primary (1^0^)/ recurrent lesion/multiple lesions** | **Recurrence during follow-up (%)** | **Lesion Site** |
| --- | --- | --- | --- | --- | --- | --- | --- |
| Barysch *et al* ^32^ | 100% | Good (54.4%) ; Moderate (18.3%); Poor (8.9%)^a^ | Mean: 3.5cm | 1 patient only | 1^o^: 98.9% | 13% | Head & neck (86.7%); all sites included |
| Piccinno *et al* ^30^ | 100%; punch biopsy | N/A^a^ | <2cm (38.3%)  ≥2≤4 (57.4%)  N/A (4.3%) | N/A | 1^0^: 74.5%  Recurrent / Salvage: 21.3%  Adjuvant: 4.2% | 15% at 5 & 10 years | Vermillion lip only |
| Moloney *et al* ^23^ | N/A | N/A | Size: 1cm (IQR 1, 1.5).  Stages:  Epidermis (1.4%)  ≤2cm (65.4%)  > 2cm (13.2%) | N/A | N/A | 0.75% | Head & Neck (66.8%) ; all sites included |
| Zagrodnik *et al* ^29^ | 100% | BCC study only: Nodular (58.9%); Superficial (14.3%); Sclerosing (26.8%) | N/A | N/A | All 1^0^ tumours; 20 patients with > 1 lesion | 15.8% | Head & trunk (94.8%) |
| Thom *et al* ^31^ | 53% overall  24% of recurrent lesions | Limited information (of 369 lesions, 4 Infiltrative, 2 Aggressive, 7 Perineural invasion | N/A | N/A | 1^0^ :1 only 77.2%; remainder > 1  Recurrent: 14,6%  Adjuvant: 9.8% | 1.27% (incomplete follow up in 91%) | Head (89.9%) |
| Tran *et al* ^27^ | N/A | N/A | Mean: 1.3 cm  < 2 cm: 78.1% (BCC); 73.2% (SCC)  ≥2<4: 19.3% (BCC) ; 24.4% (SCC) | N/A | N/A | 0.32% | Head & neck (64.3%) ; also trunk & extremities |
| Yu *et al* ^26^ | 100% | N/A | Median 1 cm ;  36.8% SCCIS ^c^;  ≤2 cm: 57.9%  > 2cm 5.3% | N/A | 1^0^  Multiple lesions in 40% | 0.75% | N/A |
| Roth *et al* ^24^ | N/A | BCC types: nodular 49% infiltrative 16%, superficial 11%, mixed superficial / infiltrative 11%.  SCC: in situ 42%; well differentiated 20%; KA ^b^ 10% | Mean 1.56 cm  (range: 0.5-6.5 cm)  < 2cm: 70.5%  >2cm : 29.5% | N/A | 1^o^: 98.7%  Multiple lesions in 29% | 0.79% | Head & neck (67%). In addition, extremities and trunk |
| Roth *et al* ^33^ | 100% | N/A | Mean 1.087 cm  (range 0.4-3 cm)  < 1cm: 32%  1-2 cm: 59%  >2cm : 9% | N/A | 1^o^ :100%  Multiple lesions in 32.4% | 2.6% | Analysis confined to lower extremities. |
| Cognetta et al ^28^ | 100% | Described by nodular vs superficial for BCC & In situ vs Invasive for SCC | N/A | N/A | 1^o^ :100%;  Multiple lesions in ~ 40% | 2.6% | Predominately head & neck |
| Locke *et al* ^25^ | 100% | Only BCC & SCC defined | Defined by:^€^  < 1cm: > 90%  1.1-2 cm: < 10%  2.1-5 cm: < 5%  > 5 cm: < 1% | N/A | 1^o^: 69.6%  Recurrent: 31.4%  Multiple lesions included. | Overall 11% ;  7% in 1^o^ cases; 20% for recurrent lesions | Predominantly head & neck |
| Madorsky *et al* ^21^ | 100% | < 5mm thickness on clinical exam | Selected to be <6cm | N/A | Recurrent lesions 11.4%. Multiple lesions included. | 1.6% | Head & neck (80%) |

^a^: N/A = not available in publication

^b^: KA =keratoacanthoma

^c^: SCCIS= squamous cell carcinoma in situ
