# Supplementary material for: Recurrence of keratinocyte cancers after superficial radiation therapy
Source: Skin Health Dis. 2026 May 14;6(4):369–74. doi: 10.1093/skinhd/vzag047 (PMC13425004; doi:10.1093/skinhd/vzag047)
Supplement: vzag047_Supplementary_Data [file vzag047_supplementary_data.zip › Table S3.docx]

**Table S3 Recurrence rates of SRT for the treatment of keratinocyte cancers**

| **Sources** | **Predominant site of lesion** | **Follow-up Range (months)** | **Follow-up (months)** | **Overall Recurrence Rate (%)** | **Recurrence Rate of SCCs (%)** | **Recurrence Rate of BCCs (%)** |
| --- | --- | --- | --- | --- | --- | --- |
| Barysch *et al* ^32^ | Head and Neck | 12-120 | 57 (mean) | 13 | 13 | N/A |
| Piccinno *et al* ^30^ | Lip | 1-187 | 39 (mean) | 8.5 | 8.57 | 8.3 |
| Moloney *et al* ^23^ | Head and Neck | max: 66 | 25.06 (mean) | 0.92 | 0.76 | 1.03 |
| Zagrodnik *et al* ^29^ | Head and Neck | 12-60 | 48 (median) | 12.57 | N/A | 12.57 |
| Thom *et al* ^31^ | Head and Neck | 0-12 | 1.1 (median) | 0.61 | 1.09 | 0.42 |
| Tran *et al* ^27^ | Head and Neck | 0–64 | 15.07 (mean) | 0.40 | 0.84^a^ | 0.76^a^ |
| Yu *et al* ^26^ | N/A | 0-31 | 16.23 (mean) | 0.80 | 0 | 1.49 |
| Roth *et al* ^24^ | Head and Neck | 1-85 | 36.5 (mean) | 0.79 | 0.61 | 0.89 |
| Roth *et al* ^33^ | Lower Extremities | 0-69 | 39.10 (mean) | 3 | 2.65 | 2.63 |
| Cognetta et al ^28^ | Head and Neck | 1-120 | 31.5 (mean) | 2.60 | 2.31 | 3.09 |
| Locke *et al* ^25^ | Head and Neck | 24-288 | 69.6 (mean) | 11 | 20 | 8 |
| Madorsky *et al* ^21^ | Head and Neck | 0.5-108 | 60 (mean) | 1.6 | N/A^b^ | N/A^b^ |

^a^: Based on Kaplan-Meier data.

^b^: Specific recurrence data for BCCs and SCCs was not provided.
